# Supplementary material for: Usefulness of Systemic Venous Ultrasound Protocols in the Prognosis of Heart Failure Patients: Results from a Prospective Multicentric Study
Source: J Clin Med. 2023 Feb 6;12(4):1281. doi: 10.3390/jcm12041281 (PMC9966251; doi:10.3390/jcm12041281)
Supplement: Supplementary file 1 [file jcm-12-01281-s001.zip › jcm-2165326-supplementary.pdf]

Journal of
Clinical Medicine

MDPI

Supplementary Materials: ROC analysis from all ultrasound parameters

1. ROC CURVE TO PREDICT MORTALITY (GLOBAL):

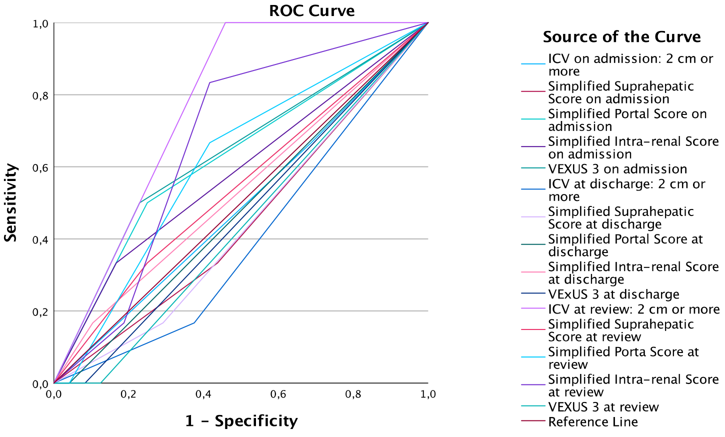

Diagonal segments are produced by ties.

Test Result Variable(s)	Area	Std. Error[a]	Asymptotic Sig.[b]	Asymptotic 95% Confidence Interval	
				Lower Bound	Upper Bound
ICV on admission: 2 cm or more	,490	,127	,934	,241	,738
Simplified Suprahepatic Score on admission	,448	,123	,680	,207	,689
Simplified Portal Score on admission	,625	,128	,322	,374	,876
Simplified Intra-renal Score on admission	,583	,132	,509	,324	,843
VEXUS 3 on admission	,635	,129	,283	,383	,887
ICV at discharge: 2 cm or more	,396	,114	,409	,173	,618
Simplified Suprahepatic Score at discharge	,438	,118	,620	,205	,670
Simplified Portal Score at discharge	,479	,122	,869	,240	,719
Simplified Intra-renal Score at discharge	,531	,130	,804	,276	,786
VExUS 3 at discharge	,458	,118	,741	,227	,690
ICV at review: 2 cm or more	,771	,072	,032	,629	,912
Simplified Suprahepatic Score at review	,542	,129	,741	,289	,795
Simplified Porta Score at review	,611	,117	,378	,383	,839
Simplified Intra-renal Score at review	,665	,098	,191	,473	,857
VEXUS 3 at review	,438	,114	,620	,214	,661

2. ROC CURVE TO PREDICT MORTALITY DURING ADMISSION:

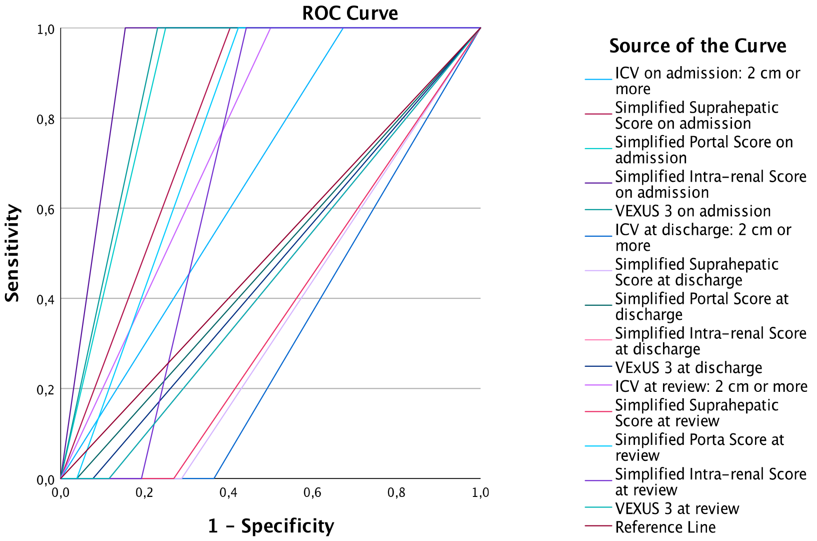

Diagonal segments are produced by ties.

Test Result Variable(s)	Area	Std. Error[a]	Asymptotic Sig.[b]	Asymptotic 95% Confidence Interval Lower Bound	Asymptotic 95% Confidence Interval Upper Bound
ICV on admission: 2 cm or more	,663	,147	,436	,375	,952
Simplified Suprahepatic Score on admission	,798	,095	,156	,611	,985
Simplified Portal Score on admission	,875	,065	,074	,747	1,000
Simplified Intra-renal Score on admission	,923	,046	,044	,834	1,000
VEXUS 3 on admission	,885	,061	,067	,764	1,000
ICV at discharge: 2 cm or more	,317	,140	,384	,043	,592
Simplified Suprahepatic Score at discharge	,356	,155	,492	,052	,659
Simplified Portal Score at discharge	,481	,203	,927	,084	,878
Simplified Intra-renal Score at discharge	,442	,188	,783	,074	,811
VExUS 3 at discharge	,462	,195	,855	,079	,844
ICV at review: 2 cm or more	,750	,114	,234	,526	,974
Simplified Suprahepatic Score at review	,365	,158	,521	,055	,676
Simplified Porta Score at review	,769	,094	,200	,585	,954
Simplified Intra-renal Score at review	,683	,079	,384	,527	,838
VEXUS 3 at review	,442	,188	,783	,074	,811

3. ROC CURVE TO PREDICT HEART FAILURE RELATED MORTALITY:

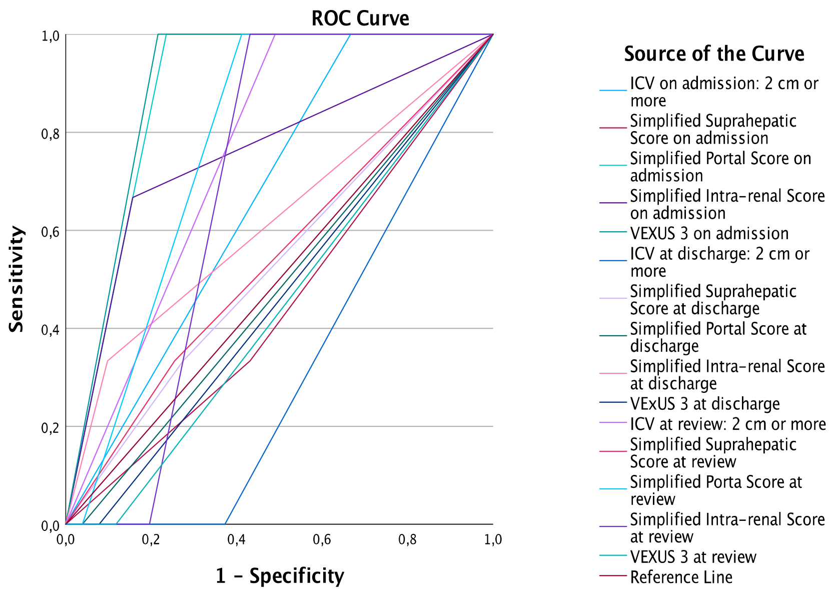

Diagonal segments are produced by ties.

Test Result Variable(s)	Area	Std. Error[a]	Asymptotic Sig.[b]	Asymptotic 95% Confidence Interval	
				Lower Bound	Upper Bound
ICV on admission: 2 cm or more	,667	,123	,336	,427	,907
Simplified Suprahepatic Score on admission	,451	,169	,777	,120	,782
Simplified Portal Score on admission	,882	,055	,027	,774	,990
Simplified Intra-renal Score on admission	,755	,164	,141	,434	1,000
VEXUS 3 on admission	,892	,052	,023	,791	,994
ICV at discharge: 2 cm or more	,314	,117	,282	,085	,542
Simplified Suprahepatic Score at discharge	,529	,176	,865	,184	,875
Simplified Portal Score at discharge	,480	,167	,910	,153	,808
Simplified Intra-renal Score at discharge	,618	,188	,497	,249	,986
VExUS 3 at discharge	,461	,161	,821	,145	,777
ICV at review: 2 cm or more	,755	,095	,141	,568	,942
Simplified Suprahepatic Score at review	,539	,177	,821	,192	,887
Simplified Porta Score at review	,775	,080	,113	,618	,931
Simplified Intra-renal Score at review	,686	,072	,282	,545	,828
VEXUS 3 at review	,441	,155	,734	,137	,746

4. ROC CURVE TO PREDICT RE-ADMISSION:

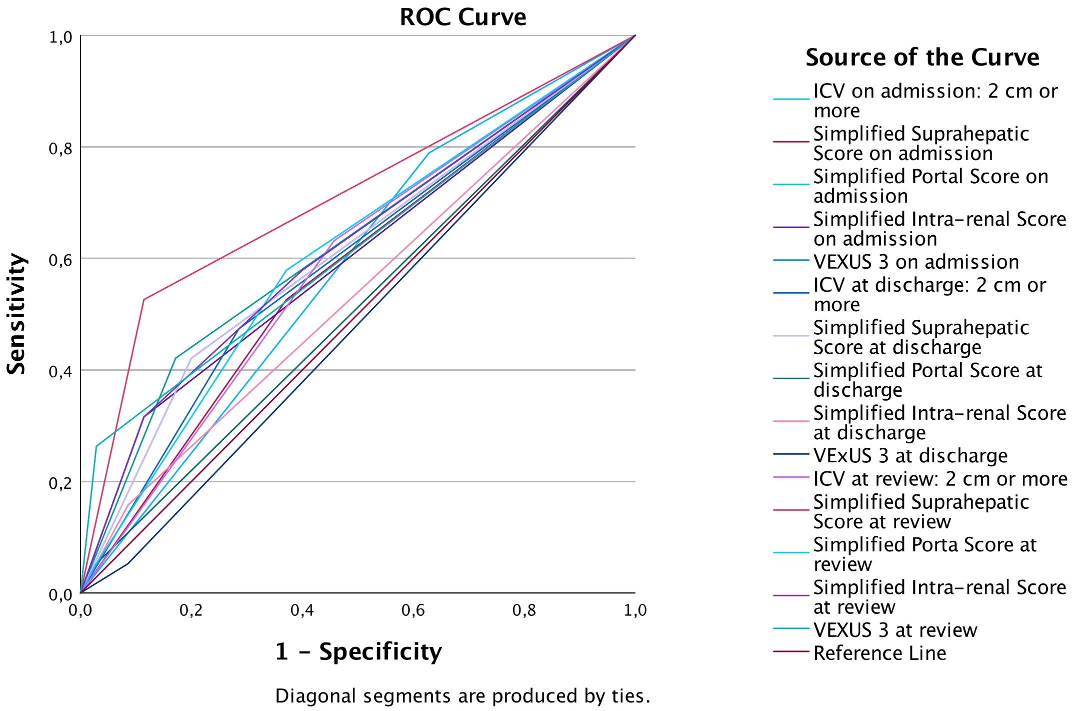

Test Result Variable(s)	Area	Std. Error[a]	Asymptotic Sig.[b]	Asymptotic 95% Confidence Interval	
				Lower Bound	Upper Bound
ICV on admission: 2 cm or more	,580	,080	,333	,423	,738
Simplified Suprahepatic Score on admission	,577	,082	,351	,416	,739
Simplified Portal Score on admission	,611	,083	,183	,448	,773
Simplified Intra-renal Score on admission	,601	,084	,225	,436	,766
VEXUS 3 on admission	,625	,083	,133	,462	,787
ICV at discharge: 2 cm or more	,594	,083	,258	,432	,756
Simplified Suprahepatic Score at discharge	,611	,083	,183	,448	,773
Simplified Portal Score at discharge	,512	,084	,885	,348	,676
Simplified Intra-renal Score at discharge	,536	,084	,664	,371	,701
VExUS 3 at discharge	,483	,082	,842	,322	,645
ICV at review: 2 cm or more	,587	,081	,293	,428	,747
Simplified Suprahepatic Score at review	,706	,079	,013	,550	,862
Simplified Porta Score at review	,605	,082	,205	,445	,765
Simplified Intra-renal Score at review	,620	,083	,150	,457	,782
VEXUS 3 at review	,617	,085	,158	,451	,783

5. ROC CURVE TO PREDICT RE-ADMISSION DURING THE FIRST MONTH:

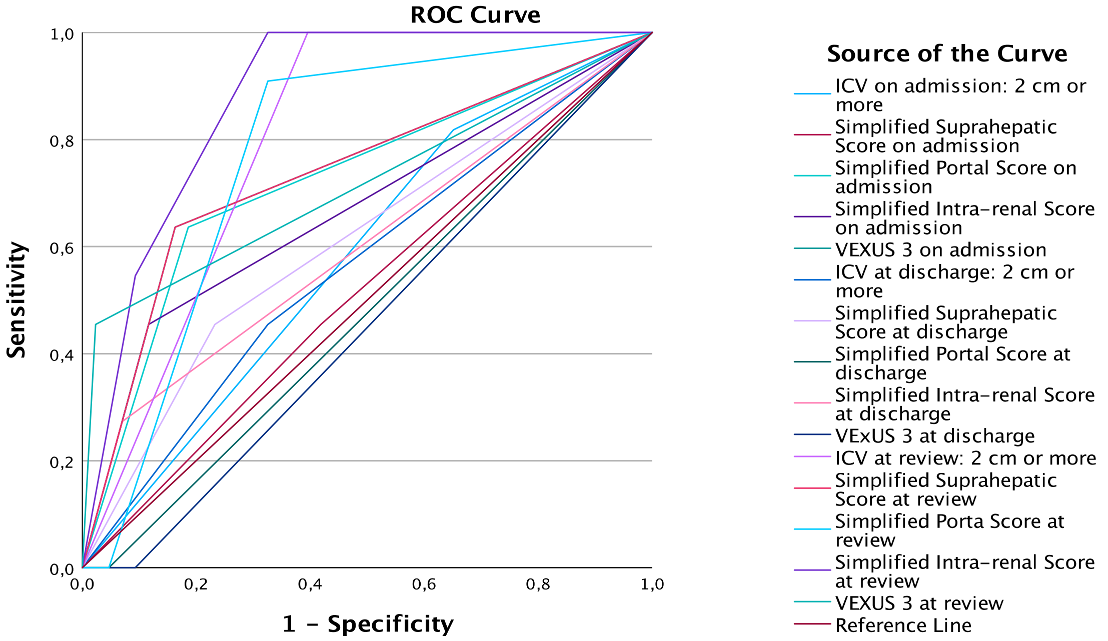

Diagonal segments are produced by ties.

Test Result Variable(s)	Area	Std. Error[a]	Asymptotic Sig.[b]	Asymptotic 95% Confidence Interval	
				Lower Bound	Upper Bound
ICV on admission: 2 cm or more	,584	,092	,396	,402	,765
Simplified Suprahepatic Score on admission	,518	,099	,855	,325	,711
Simplified Portal Score on admission	,725	,093	,022	,543	,907
Simplified Intra-renal Score on admission	,669	,101	,086	,471	,867
VEXUS 3 on admission	,737	,093	,016	,555	,918
ICV at discharge: 2 cm or more	,564	,099	,512	,370	,759
Simplified Suprahepatic Score at discharge	,611	,100	,260	,415	,807
Simplified Portal Score at discharge	,477	,096	,813	,289	,664
Simplified Intra-renal Score at discharge	,601	,104	,303	,398	,805
VExUS 3 at discharge	,453	,093	,637	,271	,636
ICV at review: 2 cm or more	,802	,059	,002	,687	,917
Simplified Suprahepatic Score at review	,737	,093	,016	,555	,918
Simplified Porta Score at review	,771	,070	,006	,633	,908
Simplified Intra-renal Score at review	,879	,046	,000	,789	,970
VEXUS 3 at review	,716	,102	,028	,516	,916

6. ROC CURVE TO PREDICT HEART FAILURE RE-ADMISSION:

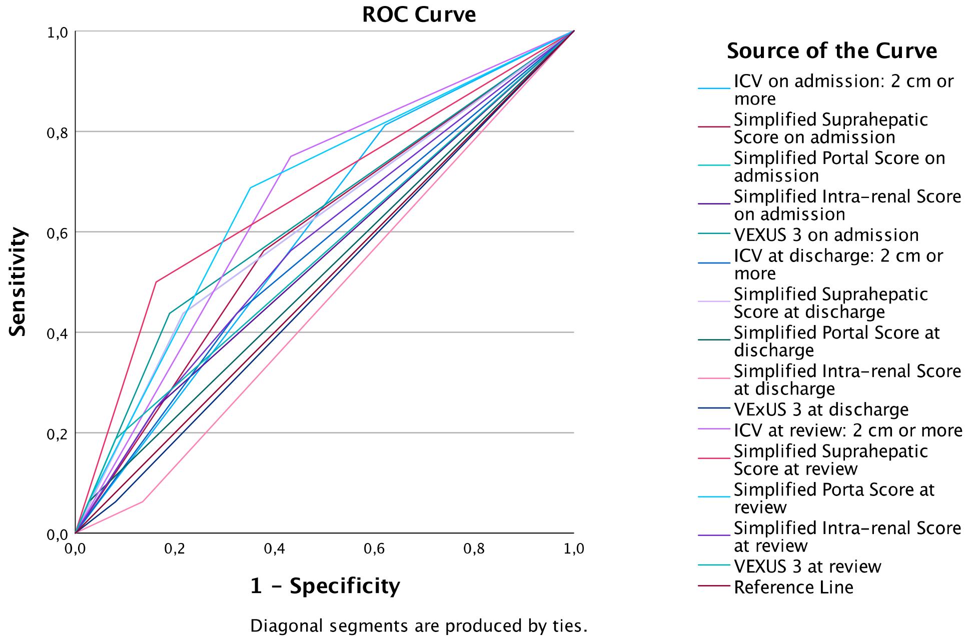

Test Result Variable(s)	Area	Std. Error[a]	Asymptotic Sig.[b]	Asymptotic 95% Confidence Interval	
				Lower Bound	Upper Bound
ICV on admission: 2 cm or more	,595	,083	,274	,433	,757
Simplified Suprahepatic Score on admission	,592	,086	,291	,423	,761
Simplified Portal Score on admission	,611	,088	,204	,439	,782
Simplified Intra-renal Score on admission	,544	,089	,614	,370	,718
VEXUS 3 on admission	,624	,088	,154	,452	,796
ICV at discharge: 2 cm or more	,557	,088	,516	,385	,728
Simplified Suprahepatic Score at discharge	,611	,088	,204	,439	,782
Simplified Portal Score at discharge	,518	,088	,839	,345	,691
Simplified Intra-renal Score at discharge	,464	,085	,677	,297	,630
VExUS 3 at discharge	,491	,087	,915	,321	,660
ICV at review: 2 cm or more	,659	,081	,069	,501	,817
Simplified Suprahepatic Score at review	,669	,086	,053	,500	,838
Simplified Porta Score at review	,670	,082	,052	,510	,830
Simplified Intra-renal Score at review	,573	,087	,399	,403	,744
VEXUS 3 at review	,553	,089	,542	,378	,728
